# Reducing sugary drink intake through youth empowerment: results from a pilot-site randomized study

**DOI:** 10.1186/s12966-019-0819-0

**Published:** 2019-07-30

**Authors:** Monica L. Wang, Marisa Otis, Milagros C. Rosal, Christina F. Griecci, Stephenie C. Lemon

**Affiliations:** 1 Department of Community Health Sciences, Boston University School of Public Health, 801 Massachusetts Avenue, Crosstown Center 4th floor, Boston, MA 02118 USA; 2 Department of Preventive and Behavioral Medicine, University of Massachusetts Medical School, 55 North Lake Avenue, Worcester, MA 01655 USA; 3 Tufts University Friedman School of Nutrition Science and Policy, 150 Harrison Avenue, Boston, MA 02111 USA

**Keywords:** Community-based intervention, Sugar-sweetened beverage consumption, Water consumption, Childhood obesity, Youth empowerment

## Abstract

**Background:**

Efficacious strategies to reduce sugar-sweetened beverage (SSB) consumption among youth are needed. This pilot study assessed the feasibility and preliminary efficacy of a community-based youth empowerment intervention to reduce SSB consumption and obesity risk among a low-income, ethnically diverse sample of youth.

**Methods:**

The H_2_GO! intervention was pilot-tested in an afterschool setting (Boys and Girls Clubs (BGC)) in Massachusetts, USA. One site was randomized to receive the intervention; the other site received standard programming. Youth ages 9–12 years and their parents/caregivers were eligible to participate. A total of *N* = 110 parent-child pairs (*N* = 55 parent-child pairs per site) were recruited. The 6-week intervention consisted of group-based weekly sessions delivered by trained BGC staff and youth-led activities that engaged parents. Child outcomes included self-reported SSB and water intake and measured body mass index z scores (zBMI). Parent outcomes included self-reported SSB and water intake, SSB purchasing, and availability of SSBs at home. Outcomes were measured at baseline, 2 months, and 6 months. Generalized linear and logistic regression models were used to estimate intervention effects over time.

**Results:**

The final analytic study sample consisted of 100 child participants (38% Black, 20% Hispanic, 13% White, 12% Multiracial, 11% Asian) and 87 parent participants (78.2% female; 78.2% reporting eligibility for the free-or-reduced price lunch program). 6-month retention rates were ≥ 82%. Intervention attendance rates among intervention child participants (*N* = 51) averaged 78.1% (SD = 10.3). Over half (56.0%) of child participants were overweight or obese at baseline. Relative to the comparison site, intervention site child participants had decreased SSB intake (β = − 1.64; 95% CI: 2.52, − 0.76), increased water intake (β = 1.31; 95% CI: 0.38, 2.23), and decreased zBMI (− 0.23 units; 95% CI: − 0.31, − 0.14) over 6 months (*p* < 0.001). Intervention parent participants also reported decreased SSB intake (β = − 1.76; 95% CI: − 2.56, − 0.96) and increased water intake (β = 1.75; 95% CI: 1.11, 2.40) than comparison parent participants at 6 months (*p* < 0.001).

**Conclusions:**

Findings demonstrate the potential of a youth empowerment intervention on reducing SSB intake and zBMI among a diverse sample. Findings will guide a larger cluster-randomized controlled trial to test intervention efficacy on preventing childhood obesity, as well as inform future interventions that aim to target additional diet and physical activity behaviors through youth empowerment.

**Trial registration:**

ClinicalTrials.gov NCT02890056. Registered 31 August 2016.

**Electronic supplementary material:**

The online version of this article (10.1186/s12966-019-0819-0) contains supplementary material, which is available to authorized users.

## Background

The positive association between sugar-sweetened beverage (SSB) consumption and childhood obesity is well-established [1–8]. SSBs are a primary source of added sugars [6] and a leading source of nutrient poor energy [9–11] in children’s diet [12]. Despite recent declines, SSB consumption among early and pre-adolescent youth in the U.S. remain high. Nearly two thirds (63.5%) of youth ages 6–11 years consumed 1 or more SSBs on a given day in 2013–2014 [13]. The average caloric intake from SSBs among 6–11 year old youth is 133 kcal/day among boys and 104 kcal/day among girls [14], with considerably higher SSB intake among low-income and ethnic minority (Hispanic and non-Hispanic Black) youth [13–17]. Low-income youth are also more likely to be heavy SSB consumers (≥500 kcals/day) than higher-income youth [17, 18]. Thus, decreasing SSB intake is a critical dietary target for obesity prevention, particularly among low-income and racial/ethnic minority youth who are at high obesity risk [1–3, 19, 20].

Empowerment-based approaches hold potential for catalyzing positive dietary behavior change by helping youth develop an ecological understanding of health and health behaviors and identify strategies for change that are meaningful and relevant in the context of their lived experience [21–26]. Empowerment approaches are also consistent with the developmental stage of early and pre-adolescents who are beginning to foster the development of their autonomy as it relates to decision-making about health-related issues [27]. Storytelling, or narratives, is a developmentally-appropriate strategy that can empower youth in behavior change [28] and may be particularly engaging for youth and families of color [22, 29, 30]. Youth-produced narratives and messages may also serve as a strategy to engage parents (important partners in child dietary interventions) in health intervention contexts [31, 32]. No studies to our knowledge have tested a youth empowerment approach to reduce SSB consumption among early and pre-adolescent youth.

To pilot a novel approach to reduce SSB consumption through *youth-produced narratives* as a strategy to facilitate empowerment and engage parents in intervention messages, we partnered with the Boys and Girls Clubs of America (BGCs), a national system of afterschool care for school-aged youth that reaches large segments of low-income and ethnically diverse youth populations. Using a pilot site-randomized controlled trial, we aim to assess the feasibility and efficacy of a community-based behavioral intervention (delivered through BGC sites) targeting SSB and water consumption among early and pre-adolescent youth (ages 9–12 years) and their parents/caregivers. We hypothesize that both child and parent intervention participants will demonstrate reduced SSB intake and increased water intake compared to comparison participants at 2 and 6 months follow-up.

## Methods

### Theoretical foundation

The development of the intervention for our pilot study was informed by the Social Cognitive Theory (SCT) [33, 34] and the Social-Ecological Model [35, 36]. Intervention activities were designed to target key SCT constructs (e.g., knowledge, self-efficacy, outcome expectations, perceived social norms, and behavioral capabilities) [37] related to SSB and water consumption and guide youth in youth-led activities (e.g., development of youth-produced narratives featuring target intervention messages, engaging parents via parent-child activities) as previously described [38]. The objectives of the youth-led activities were to provide opportunities for youth to engage and expose parents to intervention messages and to catalyze family change with the ultimate goal of driving increased parental support for targeted behaviors through modeling (reducing SSB intake) and shaping the home environment (limiting availability of SSBs at home).

### Study setting and population

The BGC is a national organization that provides affordable after-school programs for a large population (4.3 million annually) of school-aged youth (29% White, 27% Black, 24% Latino) from predominantly low socioeconomic backgrounds through over 4,300 sites nationwide [39]. Given the organization’s commitment to empowering youth to lead healthy lifestyles, BGCs were identified as an ideal intervention delivery setting. Two BGC sites (Worcester and Lowell) in Massachusetts, USA, selected for comparability in enrollment size and ethnic composition, participated in this pilot trial.

Parent-child pairs were recruited in-person by study staff from BGC sites using the following *child inclusion criteria:* ages 9–12 years; current member at the BGC study site; able to understand and communicate in English; able and willing to provide consent; parental/caregiver permission to participate; and no medical condition that limits ability to change beverage consumption behaviors; and *parental/caregiver inclusion criteria:* parent/caregiver to a BGC child member; 18 years or older; able to understand and communicate in English; able and willing to provide consent; and no medical condition that limits ability to change beverage consumption behaviors. Interested participants were screened for eligibility and consent and assent were completed for those eligible. Participants were recruited in waves from September–October 2016 and followed up through April 2017. Additional details on the screening and recruitment process have been published previously [38].

### Intervention development

The H_2_GO! intervention was designed to limit SSB intake (recommended guideline of 0 SSBs/day) and promote replacing SSBs with water (recommended guideline of 5–8 cups/day) among 9–12 year old youth and their parents. Intervention materials, strategies, format, and content were first pre-tested among a sample of parent-child pairs (*N* = 12) and staff members (*N* = 3) at a local BGC site (Lawrence, MA). The intervention was finalized based on youth, parent, and staff feedback and study staff observations on intervention feasibility, acceptability, and engagement. In preparation for intervention implementation, BGC staff received 5 h of training, led by the study principal investigator, which covered curriculum and protocols for each intervention session through a detailed intervention manual. BGC staff practiced protocol implementation, engaged in role play, and received feedback during mock sessions.

### Intervention components

The finalized 6-week intervention consisted of 12 group-based weekly sessions (1-h sessions twice a week) delivered by trained BGC program staff to BGC child study participants in the BGC setting during regular BGC hours (after-school on weekdays) and a culminating BGC open house event in which all BGC youth and parent members (including non-study participants) were invited to attend (Additional file 1). Intervention activities consisted of three main components:

1. BGC staff-led health sessions for youth. BGC staff trained in intervention delivery targeted youth knowledge, attitudes, and skills such as self-monitoring, goal-setting, and problem-solving related to SSB and water intake.
2. BGC staff-led narrative sessions for youth. BGC staff guided youth to produce narratives featuring behavioral messages on SSB and water intake. Narrative materials consisted of youth’s own stories and messages (building upon their lived experiences) and were developed by youth through a variety of mediums (print, audio, and video). Each week’s health session was followed by a subsequent narrative session reinforcing the same topic (see Table 1).
3. Youth-led activities empowering youth as change agents. Youth engaged parents by teaching parents knowledge and skills learned through weekly parent-child take-home activities and sharing of narrative materials produced, culminating in a youth-led BGC community open house event that included a display of narrative materials and youth-led taste tests of non-sweetened beverages. Parental exposure and participation in the study thus consisted of interacting with their children through take-home activities and attending the community open house event.

**Table 1:** Sample H_2_GO! intervention session topics and activities

BGC staff-led		Youth-led
Health Sessions	Narrative Sessions	Parent-child Activities
1. Water is Good for You! (hydration demonstration)	2. Develop print narratives to promote water intake	Teach parents information and skills learned through parallel weekly parent-child activities.
3. Re-Think Your Drink (blinded taste tests of flavored water)	4. Develop print narratives to encourage non-SSB alternatives	
5. Find the Facts (label reading, SSB measuring activity)	6. Develop print narratives to explain how to identify SSBs	Engage parents in critical dialogues on target behavioral messages through weekly sharing of narratives.
7. Explore the Corner Store (scavenger hunt of SSBs and non SSBs)	8. Develop audio narratives to explain how to identify SSBs	
9. Water, Water, Everywhere (role play skits to find ways to drink water)	10. Develop video narratives to find opportunities to drink water	Lead and participate in a culminating youth-led BGC community event featuring display of narratives and flavored water taste tests.
11. SSB Triggers (role play skits to manage SSB triggers)	12. Develop video narratives to manage SSB triggers	

Child participants received a reusable water bottle and a pictorial intervention booklet developed by the research team which included 45 pages of intervention activity worksheets, parent-child take-home activities, fun facts and quizzes, and water and SSB consumption tracking sheets. Activity worksheets were completed by participants during intervention sessions, and parent-child take-home activities were completed following each session. Sample session topics and activities are summarized in Table 1. Additional details on intervention strategies and intervention session activities have been previously described [38].

### Study design

A pilot site-randomized controlled trial was used to evaluate intervention feasibility and efficacy among two BGC sites (Worcester and Lowell, MA) (Additional files 2 and 3). One site was randomized to receive the intervention; the other site engaged in standard BGC programming unrelated to SSB consumption. To maximize feasibility of intervention delivery with respect to availability of space and staff-to-student ratio, the intervention was delivered in waves (approximately 25 child participants per wave to complete the intervention in one reserved classroom setting).

Participants completed study assessments at baseline, 2 and 6 months follow-up. Comparison group participants had study assessments scheduled to match the timing of each intervention wave cycle. The feasibility measures (e.g, fidelity checklist, focus groups questions for children and parents receiving the intervention, and interview guides for staff delivering the intervention) targeted intervention acceptability, implementation, and practicality [40] and were developed by the PI (MLW) and co-investigator (MCR) to be tailored for the intervention. Study staff observations, focus groups, and informal interviews were conducted by the PI and study staff post-intervention to evaluate study feasibility and intervention acceptability. BGC staff tracked child intervention session attendance rates and child and parent attendance rates at the culminating open house event. The PI and a study staff completed intervention fidelity checks independently through direct, in-person observation of intervention sessions using a checklist to score completion of intervention activities listed in the intervention manual (0 = did not do this activity; 1 = partially completed; 2 = completed) across each of the 12 intervention sessions (range of 7–11 activities per session); intervention fidelity scores were compared and any discrepancies were discussed until an agreement was reached between the two raters. Study protocol and procedures were approved by the Boston University Medical Center Institutional Review Board (protocol ID: H-34445).

### Measures

Child consumption of SSBs and water were assessed using items adapted from the Youth Risk Behavior Surveillance (YRBS) survey [41] and a validated youth food-frequency questionnaire [42]. The 2011 YRBS asked for the frequency (number of times) that youth drank regular soda over the past 7 days but did not ask youth to report average number of servings per day and did not include items on water consumption or other types of sugar-sweetened beverages. Using other beverage categories seen in Youth Adolescent Food Frequency Questionnaire [42] (water, other types of sugary drinks such as fruit punch, fruit drinks), youth were asked to report the number of servings of regular soda, other sugar-sweetened beverages (e.g., fruit drinks, fruit punch), and water they consumed on a typical day in the past 7 days.

Child height and weight were measured by staff using portable digital scales and stadiometers. Children were measured in a semi-private setting wearing light clothing (e.g., without shoes and heavy outer layers). Height and weight measurements were used to calculate body mass index (BMI), BMI z scores or zBMI (a measure that allows for comparing children of different ages over time as they grow [43]), and standard BMI-based weight status categories using the Centers for Disease Control and Prevention age- and sex-specific BMI-based growth trajectories with those at or below the 15th percentile defined as underweight; between the 16th and 84th percentile as healthy weight; between 85th and 94th percentile as overweight; and at or above the 95th percentile as obese [44].

BMI-related behaviors were assessed using items from the YRBS survey [45], including: frequency of fast food consumption over the past 7 days; number of servings of fruits and vegetables consumed on a typical day in the past 7 days; number of days engaged in at least 60 min of moderate-to-vigorous physical activity per day over the past 7 days; number of hours of sleep on an average school day; and number of hours of screen time (watching television, playing video/computer games, and using a computer for something unrelated to school work) on an average school day. Child self-efficacy to reduce SSB intake (4-point Likert scale: 4 = strongly agree; 1 = strongly disagree) was assessed using a study survey item. Child sociodemographics assessed included gender, age, and race/ethnicity.

Parent outcomes include parental SSB and water consumption (same items as in the child survey), purchase of SSBs for their family (yes/no), and availability of SSBs at home (yes/no) assessed via self-report surveys. Parent sociodemographics assessed included gender, age, race/ethnicity, child eligibility for the free-or-reduced priced lunch program (yes/no), highest level of education attained, income level, and employment status.

### Statistical analysis

Descriptive statistics (frequencies for categorical variables and means and standard deviations for continuous variables) were computed to examine participant baseline characteristics. Baseline comparisons between intervention and control participants were conducted using t-tests for continuous measures and chi-squared tests for categorical measures. Generalized linear and logistic mixed regression models were used to estimate 2-month and 6-month change in outcomes of interest associated with the intervention. Multivariable models controlling for covariates associated with BMI (fruit and vegetable intake, fast food intake, moderate-to-vigorous physical activity, screen time, and sleep) were used to estimate adjusted change in zBMI at 2 and 6 months. Analyses utilized an intent-to-treat approach. Data were analyzed using SAS v. 9.3.

## Results

### Study sample

**Fig. 1:**
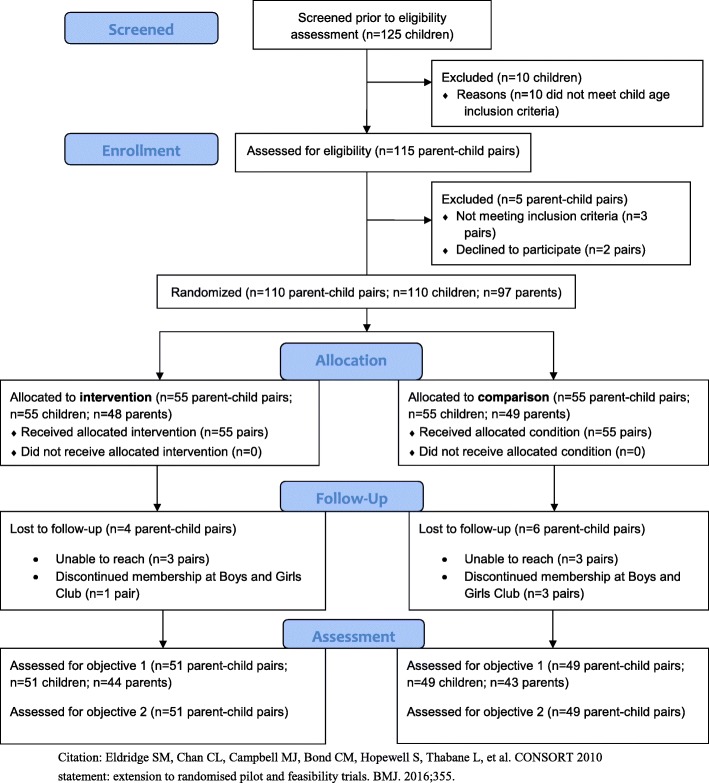
CONSORT Flow Diagram for the H_2_GO! Pilot Study. CONSORT Flowchart indicating screening, enrollment, allocation, and follow-up activities

Of the 110 child participants and 97 parent participants (*N* = 55 parent-child pairs across each of the two study sites) who were initially enrolled, those who did not have at least one follow-up assessment (*N* = 10 children; *N* = 10 parents) were excluded from analyses, resulting in a final analytic sample of 100 child participants and 87 parent participants (*N* = 51 parent-child pairs in the intervention site and *N* = 49 parent-child pairs in the comparison site; see Fig. 1). A small number of parent participants had two or more children enrolled in the study, thus accounting for the smaller number of parent vs. child participants.

### Baseline characteristics

Of the 100 child participants (38% Black, 20% Hispanic, 13% White, 12% Multiracial, 11% Asian; 5% Other), slightly less than half (46%) were female. The majority of parent participants were female (78.2%) and reported eligibility for the free-or-reduced priced lunch program (78.2%). Child participants’ mean age was 10.1 (SD = 1.0) years; parent participants’ mean age was 38.2 (SD = 8.0) years. Over half of child participants were overweight (21%) or obese (35%) at baseline. No baseline differences were observed between study sites except for race/ethnicity and gender, with the intervention site having a higher percentage of female child participants (56.9%) than the comparison site (34.7%) and a higher percentage of Black child participants (51.0%) than the comparison site (24.5%) (*p* < 0.05). On average, children consumed 2.9 (SD = 2.6) servings of SSBs and 5.4 (SD = 2.3) servings of water on a given day. Parents reported an average 2.1 (SD = 1.9) servings of SSBs and an average 5.1 (SD = 2.2) servings of water on a given day. Over half of parents reported purchasing SSBs for their family (62.1%) and availability of SSBs at home (59.8%). Additional baseline study sample characteristics are presented in Table 2.

**Table 2:** Baseline characteristics of *N* = 100 parent-child pairs (*N* = 100 children and *N* = 87 parents) participating in the H_2_GO! pilot study (2016–2017)*

Child Baseline Characteristics	Intervention (*N* = 51)	Comparison (*N* = 49)	Total (*N* = 100)	*p*-value
Gender				
Female	29 (56.9%)	17 (34.7%)	46 (46.0%)	0.026
Age (years)	10.0 (1.1)	10.2 (1.0)	10.1 (1.0)	0.48
Race				0.0012
White	4 (7.8%)	9 (18.4%)	13 (13.0%)	
Black	26 (51.0%)	12 (24.5%)	38 (38.0%)	
Hispanic/Latino	12 (23.5%)	8 (16.3%)	20 (20.0%)	
Asian	0 (0.0%)	11 (22.4%)	11 (11.0%)	
Multiracial	7 (13.7%)	5 (10.2%)	12 (12.0%)	
Other	1 (2.0%)	4 (8.12%)	5 (5.0%)	
*N = 1 missing				
Weight Status				0.52
Underweight	2 (3.9%)	3 (6.1%)	5 (5.0%)	
Healthy weight	17 (33.3%)	22 (44.9%)	39 (39.0%)	
Overweight	13 (25.5%)	8 (16.3%)	21 (21.0%)	
Obese	19 (37.3%)	16 (32.6%)	35 (35.0%)	
zBMI	1.1 (1.0)	1.0 (1.1)	1.0 (1.0)	0.39
Child Behavior				
Child SSB consumption (servings/day)	3.0 (2.7)	2.8 (2.4)	2.9 (2.6)	0.66
Child water consumption (cups/day)	5.3 (2.2)	5.5 (2.3)	5.4 (2.3)	0.73
Parent Baseline Characteristics	Intervention *N* = 44	Comparison *N* = 43	Overall N = 87	*p*-value
Gender				0.39
Female	36 (81.8%)	32 (74.4%)	68 (78.2%)	
Age (years)	38.2 (7.5)	38.1 (8.3)	38.2 (8.0)	0.95
Race				0.01
White	5 (11.4%)	4 (9.3%)	9 (10.3%)	
Black	17 (38.6%)	9 (20.9%)	26 (29.9%)	
Hispanic/Latino	19 (43.2%)	14 (32.6%)	33 (37.9%)	
Asian	1 (2.3%)	12 (27.9%)	13 (14.9%)	
Multiracial/Other	2 (4.6%)	4 (9.3%)	6 (6.9%)	
Annual Income				0.99
< $30,000	22 (50.0%)	21 (48.8%)	43 (49.4%)	
$30,000-49,999	13 (29.6%)	12 (27.9%)	25 (28.7%)	
≥ $50,000	8 (18.2%)	9 (20.9%)	17 (19.5%)	
*N = 3 missing				
Education				0.22
≤ High school degree	14 (31.8%)	20 (46.51%)	34 (39.1%)	
Some college	15 (34.1%)	12 (27.9%)	27 (31.0%)	
≥ College degree	12 (27.3%)	11 (25.6%)	23 (26.4%)	
*N = 3 missing				
Occupation				0.99
Employed full-time	22 (50.0%)	21 (48.8%)	43 (49.4%)	
Employed part-time	13 (29.6%)	12 (27.9%)	25 (28.7%)	
Other (disabled, retired, unemployed, homemaker)	8 (18.2%)	9 (20.9%)	17 (19.5%)	
*N = 2 missing				
Household eligible for the free-and-reduced-price lunch program	34 (77.3%)	34 (79.1%)	68 (78.2%)	0.34
Parent Behavior				
Purchase SSBs for family	28 (63.6%)	26 (60.5%)	54 (62.1%)	0.31
Parent SSB consumption (servings/day)	2.5 (2.2)	1.7 (1.5)	2.1 (1.9)	0.06
Parent water consumption (cups/day)	5.1 (2.0)	5.0 (2.4)	5.1 (2.2)	0.96
Home Environment				
SSBs available at home	28 (63.6%)	24 (55.8%)	52 (59.8%)	0.46

*p-values are from t-tests for continuous measures and chi-squared tests for categorical and binary measures

### Intervention fidelity and exposure

The average fidelity score of intervention delivery was 193 (SD = 5.6) (possible range 0–212 or 91.0%). The mean duration of each intervention session (intended to be 1 h) was 61.1 min (SD = 4.4). Intervention attendance rates among child participants averaged 78.1% (SD = 10.3) across the 12 intervention sessions, with 90.5% of child participants and 74.5% of parent participants attending the culminating youth-led BGC open house event. Primary reasons that children missed intervention sessions included conflicting events (e.g., sports games, health appointments) and sick days. The majority of parent participants (70.2%) reported being exposed to at least 2 out of the 3 parental intervention activities (viewing child-produced narratives; completing take-home activities with their child; and attending the BGC open house event).

### Intervention effects

Tables 3 and 4 present results from generalized linear and logistic regression mixed models estimating intervention effects associated with 2-month and 6-month change from baseline in outcomes of interest among child and parent participants (difference of the difference over time between intervention and comparison site participants). The H_2_GO! intervention was associated with 6-month reductions in servings of SSBs consumed per day (β = − 1.64; 95% CI: 2.52, − 0.76; *p* < 0.001) and 6-month increases in servings of water consumed per day (β = 1.31; 95% CI: 0.38, 2.23; *p* = 0.0063) among child participants relative to comparison site participants. Among parent participants, the intervention was associated with 6-month reductions in SSB consumption (β = − 1.76; 95% CI: − 2.56, − 0.96; *p* < 0.001) and increases in water consumption (β = 1.75; 95% CI: 1.11, 2.40; *p* < 0.001) relative to comparison parent participants. Within-group analyses among intervention parent participants indicated decreased odds of SSB availability in the home environment (OR = 0.4; 95% CI: 0.2, 0.8) at 2 months and (OR = 0.4; 95% CI: 0.2, 0.8) at 6 months, though this was not statistically significant in between-group analyses.

**Table 3:** Results from generalized linear mixed effects regression models examining change in outcomes over time associated with the H_2_GO intervention among child participants (*N* = 100)

Child Outcomes	Unadjusted				Adjusted^a^			
	Intervention effect 2-month change from baseline		Intervention effect 6-month change from baseline		Intervention effect 2-month change from baseline		Intervention effect 6-month change from baseline	
	Diff (95% CL)	*p*-value	Diff (95% CL)	*p*-value	Diff (95% CL)	*p*-value	Diff (95% CL)	*p*-value
SSB intake (servings/day)	−0.97* (−1.75, −0.20)	0.014	−1.64^b^ (−2.52, −0.76)	< 0.001	–	–	–	–
Water intake (cups/day)	1.23^b^ (0.42, 2.05)	0.0035	1.31^b^ (0.38, 2.23)	0.0063	–	–	–	–
zBMI	−0.080 ^b^ (−0.13, −0.033)	0.0010	−0.23^b^ (−0.31, − 0.14)	< 0.001	−0.072^b^ (−0.11, −0.035)	< 0.001	− 0.22^b^ (− 0.31, − 0.14)	< 0.001
Self-efficacy to reduce SSBs	0.69^b^ (0.061, 1.32)	0.032	0.50 (− 0.24, 1.24)	0.18	–	–	–	–

^a^adjusted for BMI covariates (child fruit and vegetable consumption, fast food consumption, moderate-to-vigorous physical activity, screen time, and sleep)

^b^< 0.05

**Table 4:** Results from generalized linear and logistic mixed effects regression models examining change in outcomes over time associated with the H_2_GO intervention among parent participants (*N* = 87)

	Intervention Effect 2-Month Change from Baseline		Intervention Effect 6-Month Change from Baseline	
Parent Outcomes	Diff (95% CL)	*p*-value	Diff (95% CL)	*p*-value
SSB intake (servings/day)	− 1.67^a^ (− 2.31, − 1.03)	< 0.001	− 1.76^a^ (− 2.56, − 0.96)	< 0.001
Water intake (cups/day)	1.43^a^ (0.91, 1.94)	< 0.001	1.75^a^ (1.11, 2.40)	< 0.001
	OR (95% CI)	*p*-value	OR (95% CI)	*p*-value
Purchase SSBs for family	1.10 (0.46, 2.62)	0.83	0.91 (0.38, 2.19)	0.83
SSBs available at home	0.60 (0.25, 1.41)	0.24	0.55 (0.23, 1.30)	0.17

^a^< 0.05

Improvements in child zBMI at 2 and 6 months were also observed. A two-sample t-test on 6-month change indicated a small zBMI reduction in the intervention site (− 0.05 units; SD = 0.3) vs. the control site (+ 0.19 units; SD = 0.1) (mean difference of 0.24; 95% CI: 0.14–0.34; *p* < 0.001). Our fully adjusted model controlling for child mean fruit and vegetable intake, frequency of fast food intake, moderate-to-vigorous physical activity, screen time, and sleep indicated that children in the intervention site had a 0.072 unit decrease (95% CI: − 0.11, − 0.035; *p* < 0.001) in zBMI from baseline to 2 months and a 0.22 unit decrease (95% CI: − 0.31, − 0.14; *p* < 0.001) in zBMI from baseline to 6 months relative to children in the comparison site. No significant intervention effects associated with parental SSB purchasing, SSB availability at home, or other measured child BMI-related behaviors (child fruit, vegetable, and fast food intake, physical activity, screen time, or sleep) from baseline to 6 months were observed.

### Feasibility and acceptability

Recruitment rates of study participants were high (> 95% across both study sites). 6-month retention rates ranged from 82.0–94.4% among child and parent participants. BGC staff (*N* = 3) and children reported a high enthusiasm and satisfaction with the intervention. With respect to their experiences with delivering the intervention, one staff member commented, “*It’s refreshing to see youth get excited about health curriculum. This program does it in a way that gets them actively involved through hands-on activities, empowers them to ask questions, and encourages them to be creative. I love seeing them have fun with it!”* Another staff member described the intervention manual as *“very straightforward and easy to follow...each session built on the one prior so it allowed for scaffolding and honing in one specific dietary message. At the same time, the activities encourage co-learning and youth leadership skills.”* The majority (84.3%) of child participants reporting they would refer the intervention to a friend and 76.5% who reported they would participate in a similar intervention targeting a different dietary behavior. Over half of child participants (54.9%) requested re-enrollment in the intervention if offered in the future.

Staff from the pre-testing site reported continuing to implement the intervention independently and without additional study resources, explaining that “*this kind of program fits very well within our existing Healthy Habits program and uses existing BGC resources and equipment.”* Informal interviews with BGC staff indicated that the intervention requires 1 BGC staff member to receive training and to deliver the program, with additional staff members or BGC volunteers (as available) welcome to assist in delivering the intervention. The total approximate program cost was $1,935 for one wave or cycle of the intervention or $70.36 per child. Program costs included intervention materials needed to deliver the intervention (e.g., printed intervention manuals and booklets, reusable cups and pitchers, reusable water bottles, assorted beverages and supplies for taste testing and demonstration activities, measuring cups and spoons, arts and craft supplies, and audio/visual equipment) as well as honorarium to BGC sites to recognize staff time and effort to receive training and to deliver the program ($1000/staff member per intervention wave).

## Discussion

Our pilot H_2_GO! intervention demonstrated feasibility and preliminary evidence of reducing SSB consumption and zBMI among a low-income, ethnically diverse sample of youth ages 9–12 years and their parents through BGCs, a setting that has national reach and infrastructure to support the H_2_GO! intervention. In our study, both children and parent participants had high baseline SSB consumption, averaging 2.9 servings per day among youth and 2.1 servings per day among parents. These rates exceed national recommendations and are consistent with national estimates documenting high SSB consumption among low-income and ethnic minority populations in the U.S. [13, 14]. Results from our pilot study indicated promising findings of a single-target youth empowerment intervention on reducing child and parental SSB intake, increasing child and parental water intake, and reducing child zBMI over the 6 month study period.

Improvements in beverage intake and zBMI observed in our study are consistent with prior studies of SSB interventions that have demonstrated reductions in SSB consumption and weight gain among youth over time [46–48]. Randomized trials of SSB interventions among youth, though few, have been associated with reduced zBMI growth and decreased prevalence of overweight among samples inclusive of healthy weight children [48, 49], as well as better weight control among overweight or obese youth [47, 50, 51]. However, prior SSB interventions have been limited by small sample sizes [46], inadequate representation of ethnic minorities [49], and lack of intervention sustainability and scalability (e.g., home deliveries of beverages, random assignment to receive beverages in schools) [46–48]. Our pilot H_2_GO! intervention shows promise in addressing these gaps by successfully engaging youth in changing their SSB consumption behaviors through a community-based intervention designed for delivery and dissemination through the BGC setting.

Notably, our pilot study demonstrated improvements in child zBMI with a single-target dietary focus (SSB consumption). Though most successful childhood obesity prevention interventions are multicomponent and target multiple behaviors [52–54], studies including our own demonstrate that diet is a complex, multidimensional behavior that requires substantial skills and resources to support change [55, 56]. Single-target interventions may be a more feasible approach to engage vulnerable (low-income and ethnic minority) families and provide motivation (i.e., serve as a catalyst or a gateway) to make additional behavior changes [57–60], though further evidence of such approaches are needed. Among adults, a randomized behavioral trial solely targeting SSB intake demonstrated decreased SSB intake, BMI [61], and additional spontaneous dietary improvements [57], highlighting the potential of SSB interventions as a catalyst to support more comprehensive intervention efforts. Findings from our study support calls for strategies needed to reduce SSB consumption as a critical dietary target for childhood obesity prevention, particularly among low-income and ethnic minority youth who have high SSB consumption and are at higher obesity risk [14, 62].

A distinguishing feature of the H_2_GO! intervention is the focus on empowering youth to reduce SSB consumption. Unlike traditional didactic approaches, youth empowerment-based interventions recognize the importance of youth having ownership in their decisions as well as acknowledge them as experts of their own unique experiences [21, 22]. Cultivating youth narratives or stories is one empowerment strategy that may facilitate health behavior change among ethnic minority youth [22, 28, 31, 32]. Specifically, guiding and supporting youth to create their own narratives engages participants in a transformative process by recognizing knowledge embedded within their personal stories; such participatory processes facilitate elaborate message processing and may be particularly engaging for marginalized populations, including youth of color [22, 29, 30, 63, 64]. Our study is the first, to our knowledge, to guide youth in designing their own narratives to be delivered to their parents within the context of a SSB intervention. This type of participatory process may have facilitated increased youth engagement, enhanced intervention uptake, and ultimately improved outcomes observed in our pilot study. However, additional studies that test youth narratives within the context of SSB and obesity-related interventions are needed to establish the key components and strategies that facilitate behavior change among youth.

Pilot study findings provided preliminary evidence of improvements in both child and parental outcomes related to SSB consumption through an intervention that primarily targeted youth. Over the 6-month study period, youth and parental SSB consumption decreased and water intake increased. In particular, the intervention was associated with nearly 2 servings decrease in parental SSB consumption and 2 servings increase in parental water consumption over 6 months. Changes in parental behaviors are important to support and reinforce changes in their children’s behaviors through modeling of SSB consumption [65–71] and establishing rules around food (e.g., determining which foods and beverages are available and accessible in the home environment, limiting availability of SSBs at home) [72–75]. While the intervention was not significantly associated with parental SSB purchasing or SSB availability at home, within-group analyses indicated intervention site parents were less likely to report availability of SSBs at home at 2 and 6 months. This finding is important given our prior research demonstrating that availability of SSBs at home is a strong predictor of youth SSB consumption, regardless of SSB availability in the school or neighborhood settings [76]. Overall, our pilot study results demonstrate the potential of engaging youth and parents to support decreased SSB consumption and increased water consumption through a community-based youth empowerment intervention.

### Lessons learned from pilot study

The participatory process of conducting our pilot study and refining our intervention and study protocols in tangent with input from youth, parent, and BGC staff participants generated key takeaways. Notably, we observed higher intervention uptake (e.g., increased youth engagement and attendance) during activities that were youth-led, encouraged co-learning, or invited youth to create. BGC staff also recommended that future intervention approaches more explicitly recognize youth (particularly those of color) as experts of their own lives to further enhance youth empowerment. The application of a formal youth empowerment theory (e.g., Empowerment Theory) [77] and measurement of empowerment as a mediator may strengthen the conceptualization, implementation, and evaluation of our intervention for future testing in a larger trial. With respect to retention rates, we observed that parent participants faced challenges in completing in-person study assessments in the BGC setting due to scheduling conflicts and limited time on weekdays. Additional strategies such as collecting multiple contact information from parent participants at baseline, scheduling pre-determined study assessments at dates/times convenient for participants, and providing options for online or phone follow-up assessments may improve retention rates in a community setting.

Study findings should be considered within the context of the following strengths and limitations. Strengths of this pilot study included: comparability of study sites and participants at baseline for a pilot site-randomized trial; rigorous pre-testing and refining of intervention materials and protocols based on input from BGC youth, parents, and staff prior to implementing the pilot study; high intervention fidelity, acceptability, and feasibility; and representation of an ethnically diverse sample of youth. Limitations of the study include the small number of study sites (*N* = 2) and participants (*N* = 100 parent-child pairs; *N* = 3 staff), use of survey items vs. 24-h dietary recall assessments to assess SSB and water intake, follow-up limited to 6 months, lack of data on child participant reasons for preferring to re-enroll or not re-enroll if future waves of the intervention were provided, and lack of data on the extent to which intervention fidelity was maintained at the site that continued to deliver the intervention after the study concluded.

## Conclusions

Results from our pilot study indicate preliminary efficacy and feasibility of delivering the H_2_GO! intervention through a community-based afterschool setting. Findings from our study highlight the potential of a community-based youth empowerment intervention to reduce SSB intake and prevent obesity among early and pre-adolescent youth and merit further testing of intervention efficacy through a larger cluster-randomized controlled trial.

## Additional files

Additional file 1: TIDieR Checklist. The Template for Intervention Description and Replication (TIDieR) Checklist presents information on the intervention and the location of the information within the main body of the paper. (DOCX 29 kb)

Additional file 2: CONSORT 2010 checklist. CONSORT 2010 checklist of information to include when reporting a pilot or feasibility trial. (DOCX 41 kb)

Additional file 3: CONSORT 2010 abstract checklist. CONSORT 2010 checklist of information to include when reporting a pilot or feasibility randomized trial in a journal or conference abstract. (DOCX 26 kb)

## Data Availability

The data that support the findings of this study are available from the corresponding author upon reasonable request.

## Abbreviations

BGC: Boys and Girls Clubs of America
BMI: Body mass index
SCT: Social Cognitive Theory
SSB: Sugar-sweetened beverages
YRBS: Youth Risk Behavior Surveillance
zBMI: Body mass index z score
